# Sonochemical preparation of alumina-spheres loaded with Pd nanoparticles for 2-butyne-1,4-diol semi-hydrogenation in a continuous flow microwave reactor†

**DOI:** 10.1039/c8ra00331a

**Published:** 2018-02-13

**Authors:** Emanuela Calcio Gaudino, Maela Manzoli, Diego Carnaroglio, Zhilin Wu, Giorgio Grillo, Laura Rotolo, Jonathan Medlock, Werner Bonrath, Giancarlo Cravotto

**Affiliations:** Dipartimento di Scienza e Tecnologia del Farmaco, NIS – Centre for Nanostructured Interfaces and Surfaces, University of Turin Via P. Giuria 9 10125 Turin Italy giancarlo.cravotto@unito.it; Milestone srl Via Fatebenefratelli, 1-5 Sorisole 24010 Italy; DSM Nutritional Products Ltd., Research and Development PO Box 2676 4002 Basel Switzerland

## Abstract

A novel protocol for microwave-assisted alkyne semi-hydrogenation under heterogeneous catalysis in a continuous flow reactor is reported herein. This challenging task has been accomplished using a multifaceted strategy which includes the ultrasound-assisted preparation of Pd nanoparticles (average *Ø* 3.0 ± 0.5 nm) that were synthesized on the μ-metric pores of sintered alumina spheres (*Ø* 0.8 mm) and a continuous flow reaction under H_2_ (flow rate 7.5 mL min^−1^) in a microwave reactor (counter-pressure 4.5 bar). The semi-hydrogenation of 2-butyne-1,4-diol in ethanol was chosen as a model reaction for the purposes of optimization. The high catalyst efficiency of the process, in spite of the low Pd loading (Pd content 111.15 mg kg^−1^ from ICP-MS), is due to the pivotal role of ultrasound in generating a regular distribution of Pd nanoparticles across the entire support surface. Ultrasound promotes the nucleation, rather than the growth, of crystalline Pd nanoparticles and does so within a particularly narrow Gaussian size distribution. High conversion (>90.5%) and selectivity to (*Z*)-2-butene-1,4-diol (95.20%) have been achieved at an alkyne solution flow rate of 10 mL min^−1^. The lead-free, alumina-stabilized Pd catalyst was fully characterized by TEM, HR-TEM, EDX, IR, XRPD and AAS. Highly dispersed Pd nanoparticles have proven themselves to be stable under the reaction conditions employed. The application of the method is subject to the dielectric properties of substrates and solvents, and is therefore hardly applicable to apolar alkynes. Considering the small volume of the reaction chamber, microwave-assisted flow hydrogenation has proven itself to be a safe procedure and one that is suitable for further scaling up to industrial application.

## Abstract 2

A novel protocol for microwave-assisted alkyne semi-hydrogenation under heterogeneous catalysis in a continuous flow reactor is reported herein.

## Graphical abstract

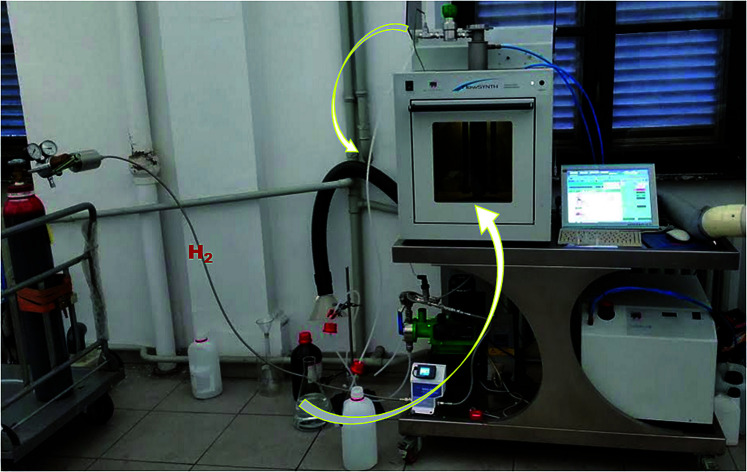

## Introduction

The development of highly efficient and environmentally benign synthetic protocols is very much a central goal of current research in chemistry. Rational designs that can be scaled up for industrial use require careful multidisciplinary analyses of safety aspects and production costs. Of the non-conventional energy sources applied to process intensification, microwave (MW) irradiation, in combination with modern flow reactors, appears well set to fulfil all necessary safety, energy saving and scalability requirements.

Heterogeneous multiphase reactions with gaseous reagents in closed MW reactors have recently been shown to be viable alternatives to conventional protocols. The catalytic hydrogenation of unsaturated organic substrates is strongly influenced by homogeneous and heterogeneous catalysis, due to the fine control of selectivity offered by advances in catalyst design.^1^ Alkynes are a versatile class of compounds that are used by the fine chemical and pharmaceutical industries despite the fact that their selective and sustainable reduction is still a significant challenge. The typical industrial approach to alkyne semi-hydrogenation involves batch hydrogenation in the presence of the Lindlar catalyst (palladium on calcium carbonate, doped with lead, satisfactory alkene selectivity),^2^ which must be removed from the product stream at the end of the reaction, in a potentially hazardous procedure. Indeed, in addition to the presence of lead, that it is well known to be toxic, the employment of large amounts of amine modifier as well as the meticulous regulation of hydrogen are usually needed to fulfil an efficient process.^3^ Such requirements imply severe disadvantages from safety, environmental and economic points of view. Therefore, routes to selective and environmentally benign alkyne reduction are highly sought after and research into new, suitable and sustainable heterogeneous catalysts is on-going.^4^ Heterogeneous catalysts offer significant advantages in reaction workup procedures and, in combination with continuous flow reactors, can furnish considerable benefits in an industrial context.^5,6^ In this frame, the possibility to work under more safe continuous-flow conditions can guarantee better catalytic performances and, at the same time, can decrease the number of processing steps.^7^ More in detail, the use of continuous flow reactors offers several advantages, such as the decrease of the purification steps and of the waste production, the reproducibility, the possibility to work with automatic operation, the decreased consumption of energy and the reduced space.^8^ All the above features contribute to the *E*-factor, that is expressed as the kg waste generated per kg product ratio,^9^ that significantly increase when using additives and other strategies in order to improve selectivity. Selective hydrogenations have already made good use of the particular properties of supported Pd nanoparticles (on active charcoal,^10^ boehmite^11^ and polymers^12^). However, the design of an ideal catalytic green system for the semi-hydrogenation of alkynes under H_2_ pressure is still a challenging task. It is worth of note that different process parameters, such as productivity per unit active metal, volume or time, absence of additives or catalyst lifetime rule the choice on continuous-flow systems in alkyne hydrogenation at large-scale with respect to conventional batch reactors.^13^ Only a limited number of alkyne semi-hydrogenations in flow reactors has been reported in the literature.^14^ These are usually carried out at very low flow rates by means of a HPLC pump (0.2 mL min), as has recently been described by Pélisson *et al.* who made use of 15 nm sized Pd nanoparticles on porous monolithic silica,^15^ providing high activity and selectivity toward *cis*-alkene thanks to the presence of Pd plane surfaces on the nanoparticles.^16^ The conversion, selectivity and stereoselectivity of the alkyne hydrogenation were tuned using the flow rates of hydrogen and the substrate solution, leading to 80% conversion and 88% selectivity toward alkenes at a flow rate of 1.0 mL min^−1^.

Moving on to catalyst production, Pd nanoparticles have been synthesized inside the hierarchical porosity of the homogeneous interconnected macropores (4 μm) and mesopores (11 nm) of silica monoliths and used as microreactors for the continuous selective hydrogenation of 3-hexyn-1-ol at room temperature. A constant conversion of 90.5% was observed, while only moderate selectivity to the *cis* isomer was achieved over a test period of 7 h. This corresponds to a 10 fold increase in productivity over Lindlar-catalyst based batch production.^17^

Even the devices used for the hydrogenation reaction have been the subject of rapid design developments as the community aims to improve the performance of catalytic hydrogenation and fine tune selectivity to the semi-hydrogenated products. The integration of a solid heterogeneous catalyst into a microreactor entails difficulties that may either be overcome by employing a micro-packed bed of powdered catalyst,^18^ or by coating the inner walls of microchannels with a thin layer of catalyst.^19^ Unfortunately, high pressure drops can occur along the reaction-channels of a powdered catalyst micro packed-bed, whereas the volume of reactor channels is barely exploited by a thin catalyst coating. Capillary microreactors offer the possibility of controlling the reagent-catalyst contact time and temperature while also removing mass and heat transfer related limitations, providing this apparatus with the possibility of achieving high selectivity.^18^ In a separate development, a continuously working quartz capillary microreactor with a diameter of 250 μm and a mesoporous-titanium-dioxide based catalytic coating with embedded Pd nanoparticles has been tested in the selective hydrogenation of 2-methyl-3-butyn-2-ol, giving 92.3% selectivity at 99.9% conversion to 2-methyl-3-buten-2-ol at 40 °C in a pure hydrogen atmosphere.^20^ Selectivity was reported to be 15.5% higher than that found in a batch reactor and the hydrogenation rate was one order of magnitude higher than observed upon reaction with the commercial Lindlar catalyst.

In an even more recent study, a micro-reactor with 4 thin layers of catalyst powder separated by commercial nylon porous butyne on milled samples of a commercial egg-shell Pd/Al_2_O_3_ catalyst.^21^ Results were compared with those obtained in a conventional fixed bed reactor in the presence of the same catalyst. However, it was reported that the catalyst pellets were employed in their original size (2.3 mm in diameter) and therefore exhibited strong diffusional effects. The micro-reactor displayed a significant increase in catalytic activity (12.5 times greater) and selectivity while also proving to be appropriate for a reliable description of intrinsic reaction kinetics, which is essential for the design of industrial scale chemical reactors. A new falling film microstructured reactor was tested in the selective hydrogenation of 2-butyne-1,4-diol to its olefinic derivative. 1.1 wt% of Pd on ZnO gave 98% selectivity at 96% conversion under optimized conditions with water as the solvent, which were close to the results of the benchmark reaction in batch mode.^22^

The wealth of possibilities currently available mean that using environmentally benign non-conventional enabling technologies to foster process intensification and combine safer protocols with cost reduction and energy savings is a promising strategy.^23^ Indeed, modern MW equipment provides easy, safe, rapid and efficient hydrogenation in the laboratory.^24^ Catalytic hydrogenation under MW irradiation has been conducted safely and quickly in open vessels,^25^ sealed reaction systems,^26^ a quartz reactor and even under 2.5 MPa of hydrogen pressure.^27^

Ultrasound (US)- and MW-assisted protocols for catalyst preparation have been extensively reported in recent decades.^28^ In particular, US has been shown to enhance nucleation,^29^ leading to narrow metal particle size distributions which are ideal in catalyst preparation,^30^ while two supported Pd catalysts have been patented (on ceria^31^ and on boehmite^32^). These lead-free Pd-catalysts have been tested on a number of substrates providing selective conversion to alkenes under conventional conditions and, with improved results, under US and MW.^33^ Results achieved with a boehmite supported Pd catalyst,^34^ have provided the necessary background for the design of a new, US-based procedure for the preparation of highly dispersed, alumina-sphere stabilized Pd nanoparticles under non-conventional conditions, which is reported herein. The catalyst has been tested in the semi-hydrogenation of 2-butyne-1,4-diol (ByD) to (*Z*)-2-butene-1,4-diol (BeD), which was chosen as the model reaction as BeD is an important chemical intermediate used in the production of vitamin B6,^35^ insecticides and fungicides,^36^ as well as being used in the paper, textile and resin industries. BeD is currently obtained commercially *via* the selective hydrogenation of 2-butyne-1,4-diol (ByD). The present study will therefore focus on the effect that MW irradiation has on ByD selective semi-hydrogenation in flow mode. Moreover, the main reaction parameters, such as substrate concentration, catalyst amount, temperature, hydrogen pressure, solvent type and volume, have all been investigated with an eye to process intensification and further scaling up. Investigations of this type are indispensable to the potential development of a pilot scale MW flow reactor for this process.

## Experimental section

### Catalyst preparation

All chemicals were purchased from Sigma-Aldrich and used without further purification. Al_2_O_3_ spheres were kindly provided by Fraunhofer ICT-IMM (Mainz, Germany). A commercial Lindlar catalyst (5% Pd on calcium carbonate, poisoned by Pb additives) was purchased from Alfa-Aesar and used as the reference catalyst.

Pd(OAc)_2_ (120 mg) was suspended in *n*-propanol (40 mL) and sonicated in a cooled cup-horn apparatus (cavitating tube Danacamerini, Turin, 19.9 kHz, 100 W) for 10 min at 30 °C. Sodium formate (72 mg) and alumina spheres (6 g) were then added to the dispersion and sonicated for 30 min at 30 °C (19.9 kHz, 100 W). The sonicated mixture was stirred for 90 min at 80 °C (under conventional heating) and finally at room temperature (overnight). The recovered solid was filtered, washed with acetone and dried under vacuum. TGA analyses were performed on the as-synthesised catalyst, and no significant weight loss was observed (see Fig. SI-1 in the ESI†), indicating that the acetone solvent was efficiently removed. Pd content of alumina-sphere was 111.15 mg kg^−1^ according to ICP-MS analysis recorded after final catalyst activation step. In this step the US prepared catalyst was flushed with ethanol (25 mL min^−1^) under MW irradiation at 65 °C for 4 min within the MW FlowSYNTH reactor recording a 12% of metal leaching which remains unchanged even by prolonging the preactivation time up to 60 min. This easy and fast procedure enabled a final Pd catalyst that showed a negligible metal leaching during the subsequent MW continuous-flow semi-hydrogenations (less than 0.1% as recorded by ICP-MS analysis after 240 min reaction).

### Catalyst characterisation

Morphological characterization was initially performed using Scanning Electron Microscopy (SEM) on a ZEISS EVO 50 XVP microscope with a LaB_6_ source, operating at 10 kV and equipped with detectors for both secondary electron and back scattered electron collection. The samples were sputtered with a gold layer (*ca.* 10 nm thickness, Bal-tec SCD050 sputter coater) prior to examination and particle size distribution was evaluated using SEM micrographs at 3000× and 5000× instrumental magnification by calculating the diameter size on more than 1000 nanoparticles. Gold coating thickness had no influence on the obtained values.

Structural characterization of the samples was carried out using a PW 3830/3020 X′ Pert Diffractometer from PANalytical with a Bragg–Brentano module (Cu Kα radiation, *λ* = 1.5406 Å) acquisition was performed in 0.02° interval steps at 5 s per step in order to provide a good signal to noise ratio.

Transmission Electron Microscopy (TEM) and High Resolution (HR)-TEM analyses were carried out on both fresh and used catalysts using a side entry Jeol JEM 3010-UHR (300 kV) microscope equipped with a LaB_6_ filament. The synthesized samples were deposited on a copper grid, coated with a lacey carbon film for analysis. All digital micrographs were acquired using a (2k × 2k)-pixel Gatan US1000 CCD camera with an OXFORD INCA instrument for atomic recognition *via* energy dispersive X-ray spectroscopy (EDX).

A statistically representative number of crystallites (>200 nanoparticles) was counted in order to provide the particle size distribution, where the mean particle diameter (*d*_m_) was calculated as; *d*_m_ = Σ*d*_*i*_*n*_*i*_/Σ*n*_*i*_, where *n*_*i*_ was the number of particles of diameter *d*_*i*_. Counting was carried out on electron micrographs obtained at a minimum of 150 000× instrument magnification, meaning that Pd particle agglomerates were clearly visible against the support.

### General methods

MW-promoted reactions were carried out in the FlowSYNTH reactor (Milestone Srl, Italy; MLS GmbH, Germany), a multimode system that operates at 2.45 GHz. This instrument was equipped with a vertical flow-through reactor, which can work up to a maximum of 200 °C temperature and 30 bar pressure and enables flow MW reactions to occur. 2-Butyne-1,4-diol (ByD) ethanol solution (0.05 w/v%) and H_2_ gas are pumped in from the bottom of the reactor (flow rate: ByD = 10 mL min^−1^ and H_2_ = 7.5 mL min^−1^; residence time: 60 s) and reaction products flow out the top into a water-cooled heat exchanger. Indeed the hydrogen flow rate has been measured with a mass flowmeter as N mL min^−1^. For the sake of simplicity, it will be reported as mL min^−1^ in the whole text. A high-performance polymer shield and a back-pressure control valve help to provide safe conditions in the PTFE-TFM flow-through reactor at all times. Moreover, integrated reactor sensors continuously monitored the internal pressure, temperature and power applied inside the reactor cavity for each reaction run and adjusted the applied MW power in real time to follow the predefined temperature profile. This system enables reactions to be scaled-up from grams to kilograms.

Aliquots (100 μL) of the solution were periodically extracted from the reaction, diluted with 900 μL chloroform and analysed using GC-MS. The analyses were carried out in a gas chromatograph Agilent 6890 (Agilent Technologies, USA) fitted with a mass detector Agilent Network 5973 using a capillary column that was 30 m long and had an i.d. of 0.25 mm and a film thickness of 0.25 mm. GC conditions were as follows; injection split of 1 : 20, injector temperature of 250 °C and detector temperature of 280 °C. The gas carrier was helium (1.2 mL min^−1^), and the temperature program proceeded from 70 °C (2 min) to 300 °C at a rate of 5 °C min^−1^.

### General reaction conditions for 2-butyne-1,4-diol semi-hydrogenation

ByD is a colourless, hygroscopic organic compound that is soluble in water and polar organic solvents. It is often applied as a model compound for the study of the selective hydrogenation of polar alkynes.^37^ The reaction was performed under MW irradiation in flow mode. The reactor cartridge (20 mL) was filled with Pd-loaded alumina spheres (6 g) and with inert materials (3–8 mm *Ø* Pyrex glass spheres 10 g) giving a residual reactor volume of 10 mL. The liquid/gas reaction mixture, the ByD ethanol solution (0.05 w/v%) and H_2_ (7.5 mL min^−1^), was pumped through the reactor (10 mL min^−1^) under MW irradiation (350 W, reaction temperature 65 °C). The FlowSYNTH work station enabled all of the key parameters to be precisely monitored. Reaction work up only involved solvent evaporation and direct GC-MS analysis (1 mg mL^−1^ EtOH/CHCl_3_ 9 : 1).

## Results and discussion

### Designing a new synthetic procedure for highly dispersed, alumina-sphere stabilized Pd nanoparticles under sonochemical conditions

**Scheme 1:**
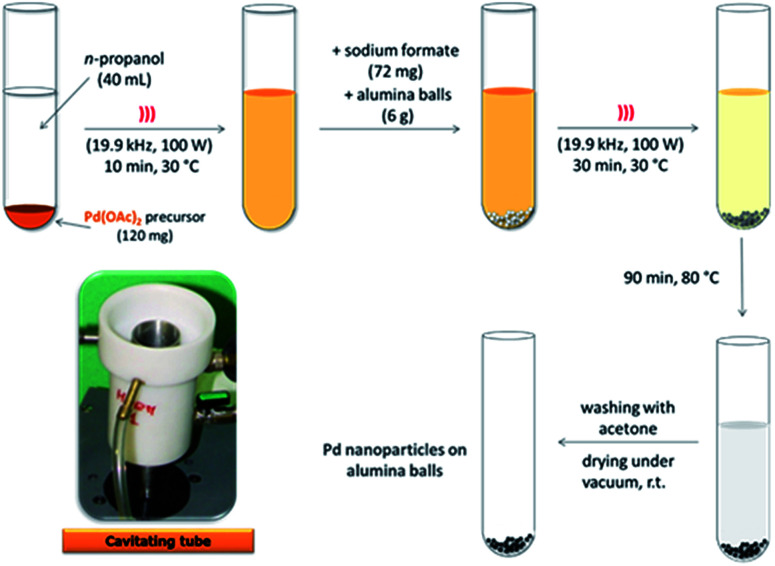
Synthetic US-assisted procedure for the preparation of highly dispersed, alumina-sphere stabilized Pd nanoparticles.

Our previous results on alkyne semi-hydrogenation in both batch and flow modes with a boehmite supported Pd catalyst,^34,38^ were the base upon which we designed a new US-assisted procedure for the production of alumina-sphere stabilized Pd nanoparticles (Scheme 1). Pd(AcO)_2_ was used as the precursor for the new heterogeneous Pd–Al_2_O_3_ catalyst which was prepared *via* the impregnation technique according to a two-step protocol.

After the initial US-assisted dispersion of Pd(OAc)_2_ in *n*-propanol (19.9 kHz, 100 W), the reduction of Pd(OAc)_2_ was performed under US (30 min, 30 °C) and in the presence of sodium formate and alumina spheres. The final impregnation step was subsequently conduced at 80 °C (90 min conventional heating). Acoustic cavitation was used as it is able to promote the rapid dispersion of solids and facilitate the formation of porous materials and nanostructures while also inhibiting particle aggregation.

### ByD selective semi-hydrogenation in flow mode under MW irradiation

The alumina-sphere stabilized Pd nanoparticles obtained (see also Catalyst section) were then tested in the selective semi-hydrogenation of ByD. It is often used as a model compound for the study of the selective hydrogenation of alkynes.^37*b*^

**Scheme 2:**
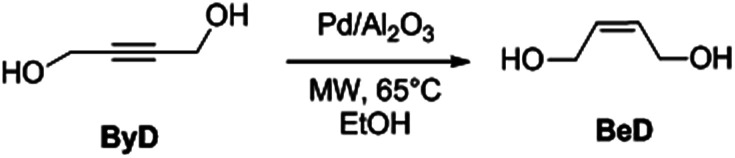
MW flow ByD semi-hydrogenation to (*Z*)-BeD on Pd/Al_2_O_3_ catalyst.

In previous works we have compared a variety of conventional conductive heating and volumetric dielectric heating protocols for the selective hydrogenation of ByD (Scheme 2).^33^

As a matter of fact, specific and selective activation of the solid catalyst surface means that dielectric heating can dramatically enhance reaction rate and selectivity.^39^

Experiments started with reactions in a batch reactor (SynthWAVE, Milestone, Italy), before continuing with flow systems (FlowSYNTH, Milestone, Italy), always with an eye to facilitating industrial scale-up. Indeed, new alumina-sphere stabilised, Pd-nanoparticle based catalytic systems can be viewed as complementary to our previously reported, Pd heterogeneous system (Pd supported on boehmite) in which ByD was selectively hydrogenated in water under MW irradiation at 90 °C in only 30 min (conv. = 100%, selec. = 92%).^33^

**Fig. 1:**
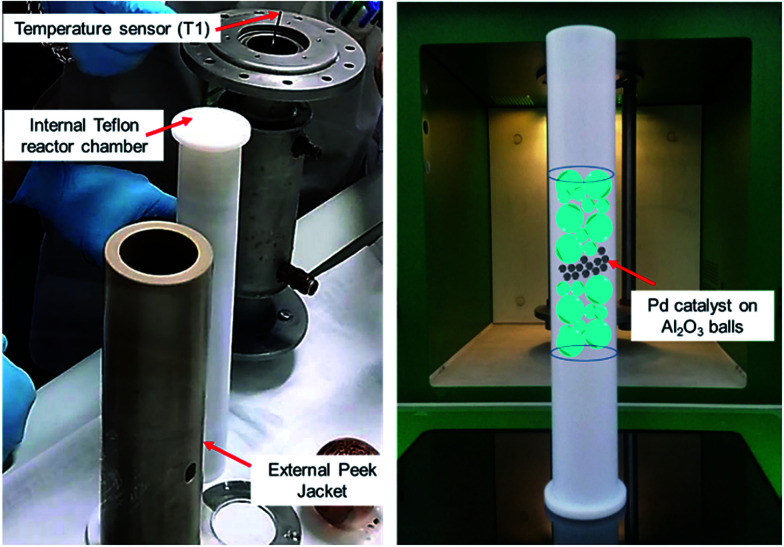
A schematic representation of the arrangement of the alumina-sphere stabilised Pd catalyst inside MW reaction chamber.

A versatile MW flow device was used to assess the performance of this catalyst under continuous flow conditions. The heterogeneous-catalyst containing reactor chamber was fixed vertically inside the multimode MW cavity. A schematic representation of the arrangement of the catalytic material inside the reaction chamber is shown in Fig. 1.

**Fig. 2:**
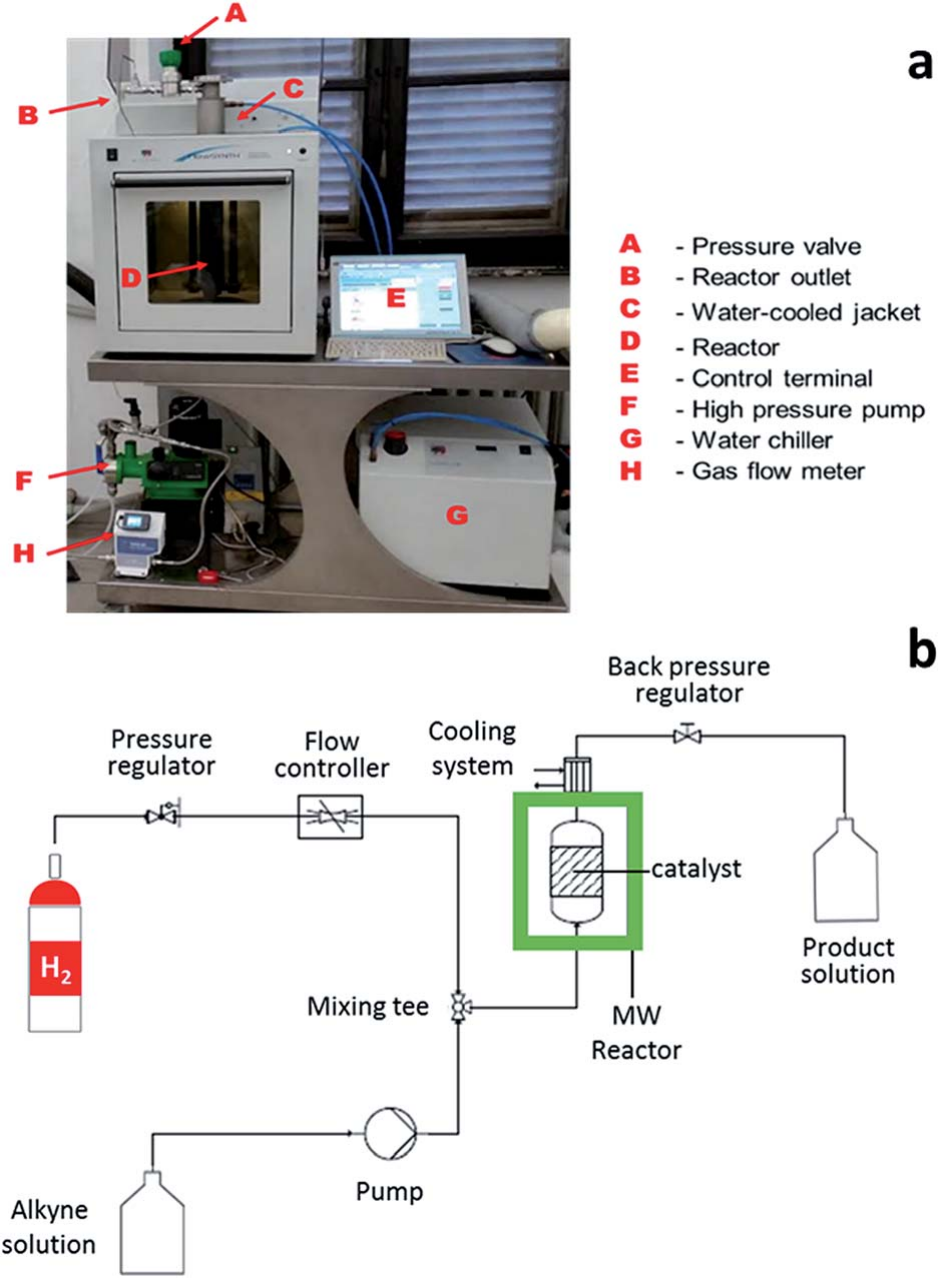
(a) MW flow reactor for alkyne semi-hydrogenation. (b) Scheme of MW continuous flow reactor setup for liquid-phase catalytic alkyne hydrogenation reaction.

In this configuration, the reactor enabled the simultaneous, rising flow of both liquids and gases to occur through the reactor, even under pressure. The flowing stream is then decompressed and the products are collected free from the catalyst. A scheme of the reactor used for alkyne semi-hydrogenation is depicted in Fig. 2a, whereas the overall MW set up is schematically reported in section b of the same figure.

The system consists of a MW lab station with a maximum power setting of 1000 W, equipped with a vertical flow-through Teflon (TFM) reactor (max volume 20 mL). The reaction product came out from the top of reactor into a water-cooled heat exchanger and sampled every 10 min for further GC-MS analyses. Reaction temperature was monitored continuously by in-line thermocouple sensors at the top of the reactor. An external touchscreen terminal was used to monitor and control all process conditions. In order to achieve best conversion and selectivity in term of (*Z*)-BeD, different residence times were explored to gain the optimized conditions of semi-hydrogenation (30–120 s), related to different ByD solution flow rates (5–20 mL min^−1^) (best result achieved for 60 s of residence time and 10 mL min^−1^ of alkyne flow).

The combination of the described MW reactor and the alumina-sphere stabilised Pd catalyst (0.012 wt% Pd), was able to selectively hydrogenate ByD under continuous flow and produce BeD as the only reaction product. It is worth noting that the selectivity (*S*) data reported in the following, were obtained as (*Z*)-2-butene-1,4-diol (BeD)/butane-1,4-diol (BaD) ratio.

**Fig. 3:**
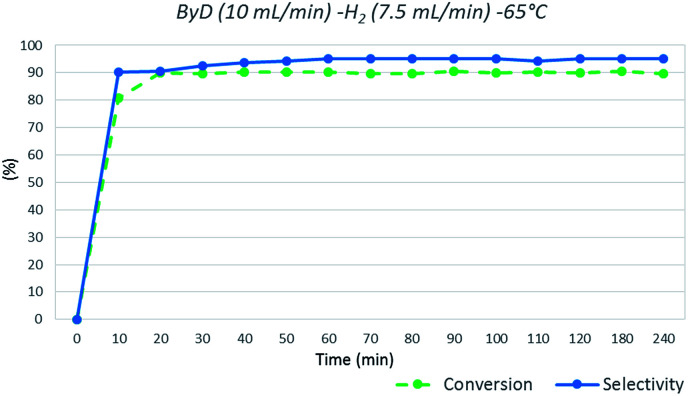
ByD flow semi-hydrogenation (10 mL min^−1^) at 65 °C and 7.5 (mL min^−1^) H_2_ flow and 4.5 bar total pressure.

In detail, a solution of ByD (ethanol solution (0.05 w/v%), flow rate = 10 mL min^−1^), flowed inside the MW reactor with hydrogen (gas flow rate = 7.5 mL min^−1^) under a total pressure of 4.5 bar, giving almost full ByD conversion (*C*: >90.5%) over 15 min of MW irradiation at 65 °C and good selectivity (*S*: 95.20%) towards BeD. The same results were produced continuously over 4 hours of MW flow hydrogenation under the same conditions, processing a total of 2.4 L of ByD solution (Fig. 3).

**Fig. 4:**
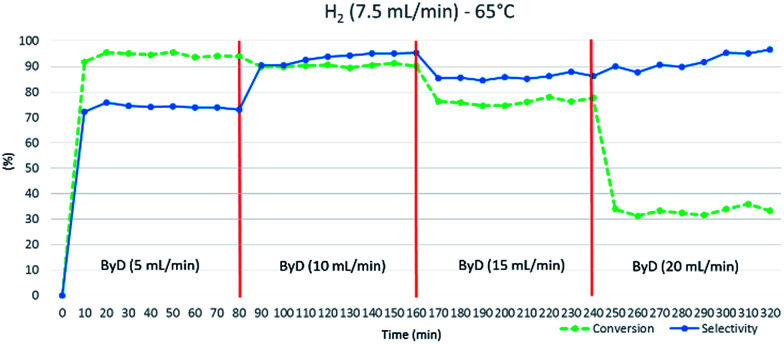
Influence of ByD flow rate on (*Z*)-BeD conversion and selectivity at 65 °C, 7.5 (mL min^−1^) H_2_ flow and 4.5 bar total pressure.

The substrate solution flow rate could be increased up to 15 mL min^−1^ with minimal conversion and selectivity losses (*C*: 76%, *S*: 87%). However, a sharp drop in conversion was observed (*C*: 33%) when the ByD flow rate was further increased (up to 20 mL min^−1^), halving residence times. 10 mL min^−1^ was thus decided to be the proper liquid flow rate (Fig. 4).

Catalyst stability and reusability were then evaluated: the Pd nanoparticle stabilization is a key factor to limit the metal leaching in solution during the semi hydrogenation (an issue of utmost importance for the reduction of metal residues in the food and pharmaceuticals manufacture industry).^5^ In this frame, the original ByD ethanol solution (0.05 w/v%) was again fluxed through the reactor over the same catalyst. Gradual catalyst deactivation was observed only after 20 L of flow reduction (substrate solution flow rate = 10 mL min^−1^; H_2_ flow = 7.5 mL min^−1^).

The alumina spheres employed in this work, due to the surface morphology, have proved to be very effective in supporting and stabilizing the Pd nanoparticles produced under sonication. It is worth of note that no metal scavengers located as downstream cartridge were required to abate metal contamination during semi-hydrogenation due to the negligible metal leaching of the used catalyst.

**Fig. 5:**
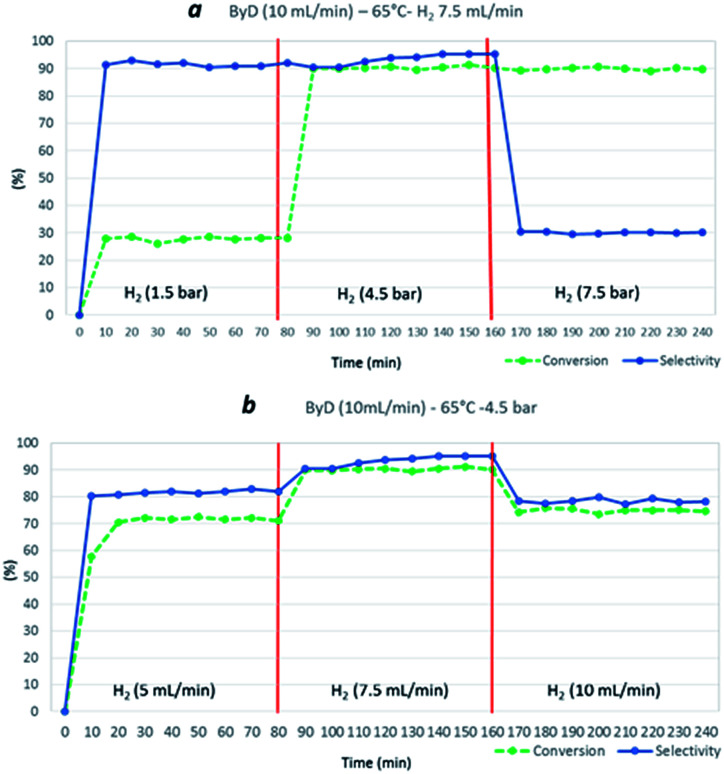
Influence of H_2_ total pressure (at a H_2_ flow rate of 7.5 mL min^−1^) (a) and influence of H_2_ flow rate (at 4.5 bar) (b) on ByD conversion and selectivity to (*Z*)-BeD at 65 °C, over a total ByD flow rate of 10 mL min^−1^.

Hydrogen flow and total H_2_ pressure also clearly influenced ByD conversion under MW. Indeed, a drastic reduction in ByD conversion was recorded (*C*: 28%) (Fig. 5a) when a ByD ethanol solution ((0.05 w/v%), flow rate = 10 mL min^−1^) was passed over the catalyst at 65 °C, together with hydrogen (gas flow 7.5 mL min^−1^) at a total pressure of 1.5 bar, instead of 4.5 bar. No ByD conversion was obtained when reactor counter-pressure was omitted or when the H_2_ flow rate was set below 5 mL min^−1^. Furthermore, total H_2_ pressure values of above 7.5 bar and total H_2_ flow rates in excess of 10 mL min^−1^ adversely affected selectivity towards (*Z*)-BeD (*S*: 78% and 30%, respectively) due to the over-hydrogenation phenomena which occur under these conditions, probably due to an enhanced residence time of the mixture inside the reactor (Fig. 5, sections a and b). At higher H_2_ flow rates (10 mL min^−1^) perhaps incomplete gas dissolution or preferential paths throw the catalyst bed negatively affected the conversion.

**Fig. 6:**
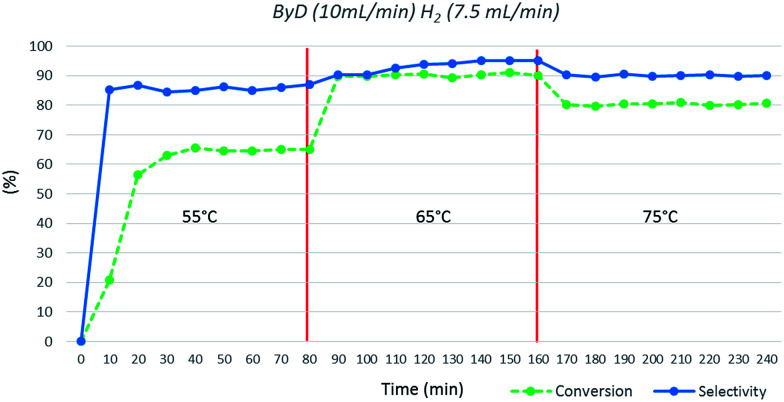
Influence of temperature on ByD conversion and selectivity to (*Z*)-BeD under MW irradiation (ByD (10 mL min^−1^) and H_2_ (7.5 mL min^−1^)) and 4.5 bar of total pressure.

Finally, the influence of temperature was investigated; very poor conversions were observed at 55 °C (*C*: 63%), whereas 65 °C appears to be the optimal choice. Moreover, conversion and selectivity toward BeD dropped (*C*: 81%; *S*: 90%) upon increasing the temperature to 75 °C (Fig. 6).

**Scheme 3:**
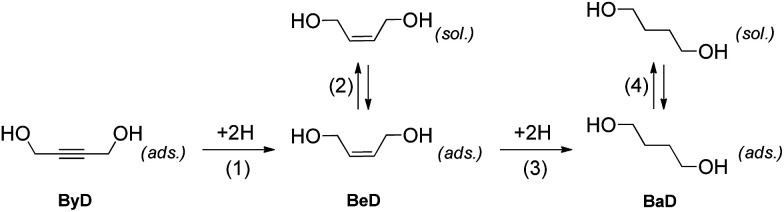


The dielectric properties of ethanol allowed for fast and selective heating to be carried out, while also having an effect on the catalyst surface and alkene adsorption–dissociation phenomena. The fact that the dissociation of the formed alkene is a key feature of reaching high conversions in semi-hydrogenations is now well-documented in the literature.^40^ The catalytic results can be qualitatively explained using the classical model proposed by Bond^16^ which can be adapted for the hydrogenation of substituted alkynes in a liquid phase, according to Scheme 3.

Selectivity in alkyne hydrogenation is governed by two alternative possible reaction paths, named (i) mechanistic and (ii) thermodynamic factors. In the mechanistic path, a chemisorbed alkene intermediate (BeD) is adsorbed onto the surface for enough time to be hydrogenated to the BaD alkane, as shown in step (3), whereas, in the thermodynamic process, the alkene can desorb and then subsequently re-adsorb in competition with the alkyne molecules, according to step (2).

In the hydrogenation of ByD under continuous MW flow conditions, the Pd catalyst showed almost full conversion and good selectivity (*C*: 90.5%; *S*: 95.2%) towards BeD, even at the early stage of the reaction when conversion is low and the re-adsorption of alkene at step (2) (thermodynamic factor) can be neglected (initial selectivity).

Both conversion and selectivity remained constant for over 4 hours, up to flow rates of 15 mL min^−1^, suggesting that the rate of alkene desorption dominates the rate of hydrogenation of the adsorbed alkene intermediate, which improves initial alkene selectivity. A further increase in flow rate, of up to 20 mL min^−1^, did not produce any improvements in catalytic activity, confirming the key role played by contact time.

The fundamental role of dielectric heating on the reaction rate was confirmed by a comparative test under optimized conditions of temperature, pressure, liquid/gas flow rate, using a preheated alkyne solution (65–67 °C) pumped in the same reactor without MW irradiation. All the samples analysed showed a negligible conversion (less than 15%).

Present results indicate that MW irradiation impact the overall catalytic process.^41^

### Catalyst characterization

According to Fig. SI-2 reported in the ESI,† the alumina-based spheres used as support for the active phase have an average size of 0.8 mm (section a) and display a reasonably regular spherical shape (section b). The EDX elemental analysis revealed that other elements (Mg, Si, Ca) are present besides Al and O, which are the principal components. X-ray powder diffraction analysis (reported in section a of Fig. SI-3†) revealed the highly crystalline nature of the spheres which are mainly made up of the Al_2_O_3_ rhombohedral phase (JCPDS file number 00-001-1296), but also include smaller amounts of orthorhombic MgSiO_3_ (JCPDS file number 00-003-0519), MgSiO_4_ (JCPDS file number 00-034-0189), monoclinic Mg and Al oxide (JCPDS file number 00-010-0238). These results are in agreement with EDX data that highlighted the presence of Mg and Si (Fig. SI-2,† section c). Moreover, no crystalline-Pd-related peaks were observed upon the addition of the metal (section b, the dashed line indicates the position of the main cubic Pd crystalline phase peak at 2 theta = 40.581), which is due to the very low amounts of metal present.

Interestingly, a closer inspection of SEM images at higher magnifications revealed that the sphere surface is indeed rough, as shown in section a of Fig. SI-4,† and that this texture is caused by the presence of small particles (section b of the same figure), that were probably sintered by the high temperature reached during spheres synthesis. The most significant sintered particle fraction displays a size of around 0.25 μm while average measured size is 0.4 ± 0.2 μm, as shown in section c.

The surface of the alumina spheres is shown, after Pd impregnation, in Fig. SI-5.† The SEM investigation demonstrated that the Pd agglomerates preferentially locate inside the recesses created by the sintered alumina particles (black zones). Moreover, the TEM measurements also confirmed the presence of Pd particle agglomerates (section b) indicating that the pre-dispersion of the Pd precursor by US led to the formation of Pd nanoparticles on the alumina microspheres.

**Fig. 7:**
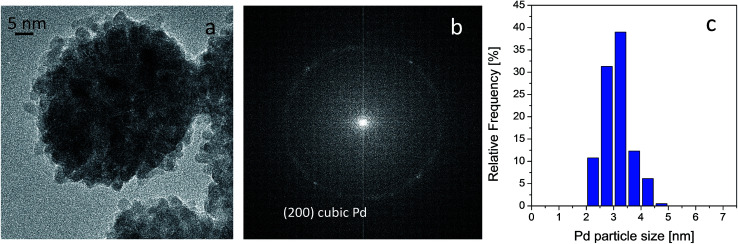
HRTEM image of the fresh Pd catalyst (a), FFT of the image reported in section a (b) and Pd particle size distribution (c). Instrumental magnification 300 000×.

HRTEM analyses were carried out on both fresh and used catalyst in order to provide further insight into the metal phase. It was found that Pd is exclusively present as spherically shaped nanoparticles, as shown in Fig. 7 section a, and that they display homogeneous size, as demonstrated by the quite narrow particle size distribution reported in section c of the same figure. Indeed, analyses provided an average diameter of 3.0 ± 0.5 nm for the spherical Pd nanoparticles. The crystalline nature of the Pd nanoparticles was also investigated by measuring the distances between the points defined by the diffracted electrons and transmitted beam in the corresponding Fourier transform (FT) of the whole range of HR-TEM micrograph images. The FT displayed a typical ring pattern of randomly oriented particles, in which some individual reflections can be also discerned (see section b of Fig. 7). Interestingly, the only reflection observed was at 0.194 nm, which is related to the (200) plane of metallic Pd in the cubic crystalline phase (JCPDS file number 00-046-1043).

### Structure–activity relationships

HR-TEM findings indicate that US promoted the formation of high purity Pd nanoparticles with uniform shape and narrow size distribution at low crystallization temperatures.

These results strongly indicate that Pd nanoparticle formation is dominated by a nucleation and growth mechanism. It can be proposed that the formation of Pd nanoparticles in the first step of the synthesis involves the creation of a complex amorphous phase network which we assume involves palladium cations creating Pd(OH)^+^ species in the *n*-propanol solution. A gel, made up of an entangled complex network of Pd hydroxide chains, is thought to take form. The second step of the synthesis involves the nucleation of primary crystalline Pd nanoparticles under sonochemical conditions.

Indeed, the microjet effect in the liquid medium generated extreme synthesis conditions (hot spots with high temperature ∼5000 K, pressure ∼20 MPa and a very high cooling rate ∼10^10^ K s^−1^),^42^ resulting in a reactant solubility enhancement which augmented the supersaturation of the reactant solution.

The nucleation process was quickened by the implosive bubble collapse, whilst the crystal growth process was somewhat hindered and slowed by the shock waves and turbulent flow created by the ultrasonic waves,^43^ which therefore promoted nucleation over grain growth to form tiny primary nanoparticles.

Almost perfectly spherical nanoparticles with a narrow size distribution were then formed in the third synthesis stage. The turbulent flow and mechanical effects created a relatively uniform reaction in the fluid medium and can improve the spherical shape of the Pd nanoparticles.^43^ High-speed microjets (over 400 km h^−1^) put pressure on the aggregated cluster from all directions meaning that crystalline nanoparticles are driven together at extremely high speeds, thus inducing effective melting at the point of impact and generating nanoparticles with a narrow size distribution. Pd nanoparticle decorated MoS_2_ nanosheets with high homogeneity and good dispersity have very recently been synthesised *via* a simple and efficient sonochemical method.^44^ The composites exhibited better electrocatalytic activity, in an oxygen electroreduction, than the commercial Pt catalyst, highlighting the effectiveness of the sonochemical approach for the facile preparation of high quality supported metal nanoparticles.

Some concepts should, perhaps, be clarified before attempting to explain the remarkable catalytic activity and selectivity displayed by these highly dispersed, crystalline and uniformly shaped Pd nanoparticles. Indeed, the crystallographic orientation of the surfaces has a strong impact on the catalytic activity. Significant differences in activity and selectivity in a number of palladium nanoparticle catalysed de-hydrogenation,^45^ and hydrogenation reactions have been reported in the literature. Both reaction classes are surface-sensitive, that is to say that the catalytic activity depends on the crystallographic orientation of the surface, *i.e.* Pd (110) is much more active than (111).^46^ In addition, also the abundance of the Pd (200) phase can influence the catalytic performance.^45^ Generally, Pd nanoparticles exhibit facets of different crystallographic orientation [*e.g.* (111) and (100)], meaning that variations in the relative abundances of the facets with particle size are able to influence overall catalytic activity, as the different facets exhibit different activity.

Kiwi-Minsker *et al.* have investigated the solution phase selective hydrogenation of alkynes over stabilized Pd nanocrystals and indicated that the semi-hydrogenation of 2-methyl-3-butyne-2-ol to 2-methyl-3-butene-2-ol is also structure sensitive.^47^ Computational and experimental modelling have been used to investigate the structure sensitivity of 2-methyl-3-butyne-2-ol hydrogenation over Pd.^48^ Further studies into supported Pd nanoparticles for acetylene gas phase hydrogenation have highlighted the fact that both Pd size and shape can significantly influence catalytic performance.^49^ In particular, acetylene hydrogenation also gave rise to ethane, in addition to ethylene, which may have either resulted from further ethylene hydrogenation or direct acetylene hydrogenation. It was observed that octahedrally shaped palladium particles were the most active as they only contain (111) facets, whereas cubically shaped Pd particles, which consists of only (100) facets, displayed low activity. Pd particles of cuboctahedral shape, which contain facets of both types, showed intermediate activity. It was found that alkynes are more strongly adsorbed onto palladium than alkenes or alkanes.^50^ This feature explains the selectivity displayed by Pd catalysts in the hydrogenation of a triple bond. Moreover, alkene adsorption onto a Pd surface should not be so strong that it prevents the reaction from proceeding sufficiently rapidly. It was found that Pd (111) was more active than Pd (100), and it was assumed that acetylene is more strongly adsorbed onto (100) faces, which would mean that hydrogenation reactions occur more slowly on (100) than on (111) faces.^50^

The metal nanoparticles with a diameter of 3.0 nm, in the present catalytic system, have a reasonable preponderance of Pd atoms in the (200) plane which are in equilibrium with the complex liquid phase, whereas Pd particles in conventional catalysts generally have a broader size distribution and, therefore, a higher concentration of atoms in the (111) plane. A doubly positive effect may result from the presence of the (200) plane: on one hand, the dissociative adsorption of H_2_, which would form highly active atomic hydrogen species that could reduce the alkyne, most likely occurs less rapidly on these Pd (200) planes than on the (111) plane. This would appear to be in agreement with literature results that show differing reactivity for the two crystalline planes in accordance with the so-called geometric effect, in which the relative amounts of different surface atoms types and particle size influence activity; on the other hand, the smaller distance of the (200) plane with respect to that of the (111) plane (1.94 Å *vs.* 2.24 Å), may mean that the alkyne is more strongly adsorbed onto the Pd (200) planes than the (100) faces, by means of a more effective interaction between Pd d orbitals and alkyne molecular orbitals (electronic effect). This may explain the high selectivity to BeD displayed by the Pd catalyst.

### On catalyst stability

**Fig. 8:**
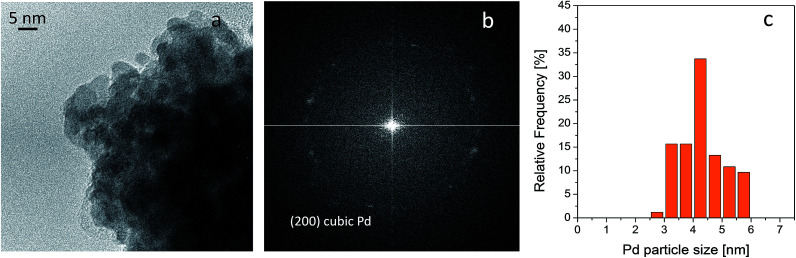
HRTEM image of used Pd catalyst (a), FFT of the image reported in section a (b) and Pd particle size distribution (c). Instrumental magnification 300 000×.

Small, crystalline Pd nanoparticles of homogeneous size were still observed on the used catalyst, as seen in Fig. 8, sections a and b. The particle size distribution indicated that very limited Pd nanoparticle agglomeration occurred during reaction. Indeed, the used catalyst was analysed after 20 L of ByD semihydrogenation under the following conditions: ByD (0.05 w/v%) = 10 mL min^−1^, H_2_ = 7.5 mL min^−1^ (4.5 bar, 65 °C). An average Pd diameter of 4.2 ± 0.7 nm which gave a stable Pd active phase despite severe reaction conditions in which continuous flow and MW irradiation were combined. In this frame, the high intrinsic surface energy meant that the highly dispersed Pd nanoparticles tended to aggregate into large particles whose stability can be expressed as follows:^51^
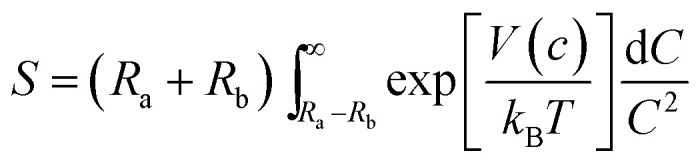
where *S* is the stability factor of the particles, *R*_a_ and *R*_b_ are the radii of the two particles, *V*(*c*) is the function of potential energy interaction, *C* is the distance between the two particles, *k*_B_ is the Boltzmann's constant (1.3806 × 10^−23^ J K^−1^) and *T* is the temperature (K). When the distance between particles is decreased to a certain extent, *i.e.* under flow and MW irradiation, short-range interactions (van der Waal's forces and the existence of an electrostatic barrier) lead to strong attraction between particles, resulting in particle aggregation and a further increase in average particle size. HR-TEM results would appear to indicate that aggregation into large particles is negligible which highlights the impressive stability of the Pd nanoparticles synthesised in this new US-assisted procedure.

### Scalability of the process

The process scalability was finally evaluated for the ByD semi-hydrogenation under the optimized reaction condition (alkyne flow rate = 10 mL min^−1^; H_2_ flow rate = 7.5 mL min^−1^; total pressure = 4.5 bar) in order to establish a proper comparison. The starting ByD amount (0.05 w/v%) in the reacting mixture was increased up to 5% and its selective MW flow hydrogenation was assessed over 60 g of the described palladium catalyst (Pd content: 111.15 mg kg^−1^ as defined by ICP-MS).

Promising results were recorded in terms of ByD conversion (*C* > 90.5%) and selectivity towards (*Z*)-BeD (*S* > 95%) over 15 min of MW irradiation (65 °C) even using more concentrated ByD solution (5%) with no detectable signs of catalyst deactivation over 4 h reaction time. These results are completely comparable with those obtained on a small scale ByD continuous flow MW semi-hydrogenation, and this confirms the effectiveness of the adopted strategy.

The intrinsic benefit of the described MW continuous flow system over conventional flow processes^5^ was proved potentially competitive on the large scale.

The use of MW gives the possibility to carry out the reaction without any selectivity promoter, which would make the process less green from an environmental point of view. Moreover, significant advantage in terms of both productivity per unit of active metal and alkyne volume to be reacted, is found when the reaction is promoted by MW.

Representative catalyst data for ByD MW semi-hydrogenation (0.05–5 w/v% ByD ethanol solution) performed in Flow SYNTH device were summarized in Table 1 in terms of mass productivity (mol gM^−1^ h^−1^) and space time yield (STY).^52^

**Table 1:** Continuous flow scalability tests on ByD microwave semi-hydrogenation

Entry^a^	Catalyst^b^ (g)	ByD^c^ (w/v%)	Conv. (%)	Sel. (%)	Yield (%)	Prod.^d^ (mol gM^−1^ h^−1^)	STY^e^ (kg L^−1^ h^−1^)
1	6	0.05	90.5	95.2	90.5	7.1	0.7 × 10^−3^
2	12	0.5	90.5	94.8	90.4	35	0.7 × 10^−2^
3	36	2.5	90.8	95.3	90.7	60	0.4 × 10^−1^
4	60	5	90.3	95.0	90.2	71	0.7 × 10^−1^

a Reaction condition: ByD flow rate = 10 mL min^−1^; H_2_ flow rate = 7.5 mL min^−1^; residence time = 60 s; temperature 65 °C.

b Activated Pd catalyst (Pd content = 111.15 mg kg^−1^ as defined by ICP-MS).

c ByD ethanol solution.

d Catalyst mass productivity (Prod.).

e Space time yield (STY).

The highest catalyst productivity (around 70 mol gM^−1^ h^−1^) on a more concentrated ByD scale for its continuous semi-hydrogenation could be justified in terms of both high accessibility of Pd sites and high permeability of the embedded activated catalyst (60 g) inside the reactor chamber (20 mL max volume) due to the MW improved heat and mass transfer effects in bigger catalyst scale.^53^

In addition, the amount of H_2_ for a residence time of 60 s under optimized conditions was calculated (28 mg of hydrogen) and corresponds to a very high alkyne : hydrogen ratio. However, this value was noticeable improved when scaling up the process. An alkyne : hydrogen ratio equal to 1.75 was obtained. This value falls in the appropriate range (1–30) for carrying out the reaction, according to the very recent review by Barbaro *et al.*^5^

Therefore the MW-assisted flow system here described could represent a favourable alternative to conventional partial-hydrogenation performed under continuous-flow.

## Conclusions

The crucial role that enabling technologies, such as US and MW, play in the design of more efficient and safer protocols for alkyne selective semi-hydrogenations in flow mode, has been fully demonstrated. High selectivity was achieved without the addition of promoters. Furthermore, a novel, yet simple, sonochemical synthesis of highly dispersed and stable Pd nanoparticles loaded on non-functionalised alumina spheres has been reported. These nanoparticles have uniform size and expose peculiar crystalline facets. Despite the physical stresses caused by dielectric heating and liquid and gas flow under pressure, Pd nanoparticle size remained almost constant. In the way of a further scaling up, preliminary tests were carried out to increase the amount of substrate in the reacting mixture up to 5% with promising results in term of mass productivity. Flow microwave technology enables reactions to be scaled-up from gram to kilogram scales, which should easily pave the way for industrial pilot reactors.

## Conflicts of interest

There are no conflicts to declare.

## Supplementary Material

RA-008-C8RA00331A-s001

## References

[cit1] Karunananda K., Mankad N. P. (2015). J. Am. Chem. Soc..

[cit2] Lindlar H. (1952). Helv. Chim. Acta.

[cit3] Jung A., Jess A., Schubert T., Schütz W. (2009). Appl. Catal., A.

[cit4] Zhao M. (2016). Chem.–Asian J..

[cit5] Moreno-Marrodan C., Liguori F., Barbaro P. (2017). Beilstein J. Org. Chem..

[cit6] Newman S. G., Jensen K. F. (2013). Green Chem..

[cit7] Lucarelli C., Vaccari A. (2011). Green Chem..

[cit8] Ricciardi R., Huskens J., Verboom W. (2015). ChemSusChem.

[cit9] (a) Sheldon R. A. , Arends I. W. C. E. and Hanefeld U., Green Chemistry and Catalysis, Wiley-VCH, Weinheim, 2007

[cit10] Joannet E., Horny C., Kiwi-Minsker L., Renken A. (2002). Chem. Eng. Sci..

[cit11] Wu Z., Rotolo L., Calcio Gaudino E., Medlock J., Bonrath W., Cravotto G. (2016). Chem. Eng. Process..

[cit12] Semagina N., Joannet E., Parra S., Sulman E., Renken A., Kiwi-Minsker L. (2005). Appl. Catal., A.

[cit13] Jiménez-González C., Poechlauer P., Broxterman Q. B., Yang B.-S., am Ende D., Baird J., Bertsch C., Hannah R. E., Dell'Orco P., Noorman H., Yee S., Reintjens R., Wells A., Massonneau V., Manley J. (2011). Org. Process Res. Dev..

[cit14] Albani D., Vilé G., Beltran Toro M. A., Kaufmann R., Mitchell S., Perez-Ramirez J. (2016). React. Chem. Eng..

[cit15] Pélisson C.-H., Nakanishi T., Zhu Y., Morisato K., Kamei T., Maeno A., Kaji H., Muroyama S., Tafu M., Kanamori K., Shimada T., Nakanishi K. (2017). ACS Appl. Mater. Interfaces.

[cit16] Bond G. C. , Metal-Catalysed Reactions of Hydrocarbons, Springer, New York, 2005

[cit17] Sachsen A., Linares N., Barbaro P., Fajula F. (2013). Dalton Trans..

[cit18] Ajmera S. K., Delattre C., Schmidt M. A., Jensen K. F. (2003). Stud. Surf. Sci. Catal..

[cit19] Haas-Santo K., Fichtner M., Schubert K. (2001). Appl. Catal., A.

[cit20] Okhlopkova L. B., Kerzhentsev M. A., Ismagilov Z. R. (2016). Kinet. Catal..

[cit21] García Colli G., Alves J. A., Martínez O. M., Barreto G. F. (2016). Chem. Eng. Process..

[cit22] Rehma T. H., Berguerand C., Ek S., Zapf R., Löb P., Nikoshvili L., Kiwi-Minsker L. (2016). Chem. Eng. J..

[cit23] Hessel V., Cravotto G., Fitzpatrick P., Patil B. S., Lang J., Bonrath W. (2013). Chem. Eng. Process..

[cit24] Schmoeger C., Gallert T., Stolle A., Ondruschka B., Bonrath W. (2011). Chem. Eng. Technol..

[cit25] Banik B. K., Barakat K. J., Wagle D. R., Manhas M. S., Bose A. K. (1999). J. Org. Chem..

[cit26] Vanier G. S. (2007). Synlett.

[cit27] Heller E., Lautenschlaeger W., Holzgrabe U. (2005). Tetrahedron Lett..

[cit28] Wu Z., Borretto E., Medlock J., Bonrath W., Cravotto G. (2014). ChemCatChem.

[cit29] Cau C., Guari Y., Chave T., Larionova J., Pochon P., Nikitenko S. I. (2013). J. Phys. Chem. C.

[cit30] Bedrane S., Descorme C., Duprez D. (2002). J. Mater. Chem..

[cit31] Cravotto G. , Bonrath W., Medlock J., Borretto E. and Wu Z., Process for preparation of Pd-on-boehmite catalytic systems for selective hydrogenation of triple bonds, PCT Int. Appl., WO 2015044411 A1 20150402, 2015

[cit32] Cravotto G. , Bonrath W., Medlock J., Borretto E. and Wu Z., Process for preparation of Pd-on-boehmite catalytic systems for selective hydrogenation of triple bonds, PCT Int. Appl., WO 2015044411 A1 20150402, 2015

[cit33] Wu Z., Rotolo L., Calcio Gaudino E., Medlock J., Bonrath W., Cravotto G. (2016). Chem. Eng. Process..

[cit34] Wu Z., Cherkasov N., Cravotto G., Borretto E., Ibhadon A. O., Medlock J., Bonrath W. (2015). ChemCatChem.

[cit35] Bonrath W., Eggersdorfer M., Netscher T. (2007). Catal. Today.

[cit36] Havis N. D., Walters D. R., Foster S. A., Martin W. P., Cook F. M., Robins D. J. (1994). Pestic. Sci..

[cit37] Telkar M., Rode C., Rane V., Jaganathan R., Chaudhari R. (2001). Appl. Catal., A.

[cit38] Wu Z., Calcio Gaudino E., Manzoli M., Martina K., Drobot M., Krtschilc U., Cravotto G. (2017). Catal. Sci. Technol..

[cit39] Kappe C. O. (2004). Angew. Chem., Int. Ed..

[cit40] Crespo-Quesada M., Grasemann M., Semagina N., Renken A., Kiwi-Minsker L. (2009). Catal. Today.

[cit41] Horikoshi S., Kamata M., Mitani T., Serpone N. (2014). Ind. Eng. Chem. Res..

[cit42] Cravotto G., Cintas P. (2006). Chem. Soc. Rev..

[cit43] Suslick K. S. (1990). Science.

[cit44] Zuo L.-X., Jiang L.-P., Zhu J.-J. (2017). Ultrason. Sonochem..

[cit45] Jeon H., Chung Y.-M. (2017). Appl. Catal., B.

[cit46] Silvestre-Albero J., Rupprechter G., Freund H. J. (2005). J. Catal..

[cit47] Crespo-Quesada M., Yarulin A., Jin M., Xia Y., Kiwi-Minsker L. (2011). J. Am. Chem. Soc..

[cit48] Presianni A., Crespo-Quesada M., Cortese R., Ferrant F., Kiwi-Minsker L., Duca D. (2014). J. Phys. Chem. C.

[cit49] Yarulin A., Crespo-Quesada R. M., Egorova E. V., Kiwi-Minsker L. (2012). Kinet. Catal..

[cit50] Bos A. N. R., Westerterp K. R. (1993). Chem. Eng. Process..

[cit51] Moghtada A., Shahrouzianfar A., Ashiri R. (2017). Adv. Powder Technol..

[cit52] Pletcher D. and Walsh F., Industrial Electrochemistry, Springer, London, 1990, p. 83

[cit53] Microwave Chemistry, ed. G. Cravotto and D. Carnaroglio, De Gruyter Graduate, Berlin, 2017

