# Supplementary material for: Sonochemical preparation of alumina-spheres loaded with Pd nanoparticles for 2-butyne-1,4-diol semi-hydrogenation in a continuous flow microwave reactor
Source: RSC Adv. 2018 Feb 13;8(13):7029–39. doi: 10.1039/c8ra00331a (PMC9078474; doi:10.1039/c8ra00331a)
Supplement: RA-008-C8RA00331A-s001 [file RA-008-C8RA00331A-s001.pdf]

Electronic Supplementary Material (ESI) for RSC Advances.
This journal is © The Royal Society of Chemistry 2018

Sonochemical preparation of alumina-sphere loaded Pd nanoparticles for 2-butyne-1,4-diol semi-hydrogenation in a continuous flow microwave reactor

Emanuela Calcio Gaudino[a], Maela Manzoli[a], Diego Carnaroglio[a,b], Zhilin Wu[a], Giorgio Grillo[a], Laura Rotolo[a], Jonathan Medlock[c], Werner Bonrath[c], Giancarlo Cravotto[a]*,

.

[a] *Dipartimento di Scienza e Tecnología del Farmaco and NIS - Centre for Nanostructured Interfaces and Surfaces, Universty of Turin, Via P. Giuria 9, I-10125 Turin, Italy.*

[b] *Milestone srl, Via Fatebenefratelli, 1-5, Sorisole 24010, Italy.*

[c] *DSM Nutritional Products Ltd., Research and Development, P.O. Box 2676, 4002 Basel, Switzerland.*

* Corresponding authors. E-mail: giancarlo.cravotto@unito.it (G. Cravotto).

Table of contents:

Experimental section **page 2**

Catalyst preparation *page 2*

Catalyst characterisation *page 2*

General methods *page 3*

General reaction conditions for 2-butyne-1,4-diol semi-hydrogenation *page 3*

TGA, SEM combined with EDX, XRPD, and TEM measurements *page 4*

Figure SI-1. page 4

Figure SI-2. page 5

Figure SI-3. page 6

Figure SI-4. page 7

Figure SI-5. page 8

References *page 8*

Experimental section

Catalyst preparation

All chemicals were purchased from Sigma-Aldrich and used without further purification. Al_2O_3 balls were kindly provided by IMM Fraunhofer Institutes (Mainz, Germany). The details of the Pd catalyst preparation and characterization are described elsewhere. A commercial Lindlar catalyst (5% Pd on calcium carbonate, poisoned by Pb additives) was purchased from Alfa-Aesar and used as a reference catalyst.

$Pd(OAc)_2$ (120 mg) was suspended in *n*-propanol (40 mL) and sonicated in a cooled cup-horn apparatus (cavitating tube Danacamerini, Turin, 19.9 kHz, 100 W) for 10 min at 30 °C. Then, sodium formate (72 mg) and alumina balls (6 g) were added to the dispersion and sonicated for 30 min at 30 °C (19.9 kHz, 100 W). The sonicated mixture was then stirred for 90 min at 80 °C (by conventional heating) and finally at room temperature (overnight). The recovered solid was filtered, washed with acetone and dried under vacuum. TGA analysis was performed on the as-synthesised catalyst, and no significant weight loss was observed (see Figure SI-5 in the Supporting Information), indicating that the acetone solvent was efficiently removed. Pd content of alumina-sphere was 111.15 mg/kg according to ICP-MS analysis recorded after final catalyst activation step. In this step the US prepared catalyst was flushed with ethanol (25 mL/min) under MW irradiation at 65° C for 4 min within the MW FlowSYNTH reactor recording a 12% of metal leaching which remains unchanged even by prolonging the preactivation time up to 60 min. This easy and fast procedure enabled a final Pd catalyst that showd a negligible metal leaching during the subsequent MW continuous-flow semi-hydrogenations (less than 0.1% as recorded by ICP-MS analysis after 240 min reaction).

Catalyst characterisation

Morphological characterization was firstly performed by Scanning Electron Microscopy (SEM) using a ZEISS EVO 50 XVP microscope with LaB_6 source, operating at 10 kV and equipped with detectors for secondary electron as well as back scattered electron collection. The samples were sputtered with a gold layer (ca. 10 nm thickness, Bal-tec SCD050 sputter coater) prior to examination and the particle size distribution was evaluated on SEM micrographs at 3,000× and 5,000× instrumental magnification by calculating the diameter size on more than 1000 nanoparticles. The gold coating thickness had no influence on the obtained values.

Structural characterization of the samples was carried out using a PW 3830/3020 X' Pert Diffractometer from PANalytical working Bragg-Brentano (Cu Kα radiation, λ=1.5406 Å). The

acquisition was performed in a 0.02° interval steps, with 5 s $step^{-1}$ to obtain a good signal to noise ratio.

Transmission Electron Microscopy (TEM) and High Resolution (HR)-TEM analyses were carried out on both fresh and used catalysts using a side entry Jeol JEM 3010-UHR (300 kV) microscope equipped with a LaB_6 filament. For analyses the synthesized samples were deposited on a copper grid, coated with a lacey carbon film. All digital micrographs were acquired by a (2k × 2k)-pixel Gatan US1000 CCD camera and with an OXFORD INCA instrument for atomic recognition via energy dispersive X-ray spectroscopy (EDX). A statistically representative number of crystallites (> than 200 nanoparticles) was counted in order to obtain the particle size distribution, where the mean particle diameter (d_m) was calculated as $d_m = \Sigma d_i n_i / \Sigma n_i$, where n_i was the number of particles of diameter d_i. The counting was carried out on electron micrographs obtained starting from 150,000× instrumental magnification, where Pd particles agglomerates well contrasted with respect to the support were clearly detected.

General methods

MW-promoted reactions were carried out in the reactor FlowSYNTH (Milestone Srl, Italy; MLS GmbH, Germany), a multimode system that operates at 2.45 GHz. This instrument equipped with a vertical flow-through reactor, which can work up to a maximum of 200 °C temperature and 30 bar pressure and enables MW flow reactions to occur. 2-Butyne-1,4-diol (ByD) ethanol solution (0.05 w/v%) and H_2 gas are pumped in from the bottom of the reactor (flow rate: ByD=10 mL/min and H_2 = 7.5 mL/min; residence time: 60s) and reaction products flow out the top into a water-cooled heat exchanger. A high-performance polymer shield and a back-pressure control valve help to provide safe conditions in the PTFE-TFM flow-through reactor at all times. Moreover, integrated reactor sensors continuously monitored the internal pressure, temperature and power applied inside the reactor cavity during all reactions run, adjusting the applied MW power in real time to follow a predefined temperature profile. This system enables reactions scale-up from grams to kilograms.

Aliquots (100 µl) of the solution were periodically extracted from the reaction, diluted with 900 µl of chloroform and analyzed using GC/MS.

GC-MS analyses were carried out in a gas chromatograph Agilent 6890 (Agilent Technologies, USA) fitted with a mass detector Agilent Network 5973 using a capillary column that was 30 m long and had an i.d. of 0.25 mm and a film thickness of 0.25 mm. GC conditions were injection split of 1:20, injector temperature of 250 °C, and detector temperature of 280 °C. The gas carrier was helium (1.2 mL min−1), and the temperature program was from 70 °C (2 min) to 300 °C at 5 °C min−1.

General reaction conditions for 2-butyne-1,4-diol semi-hydrogenation

ByD is a colourless, hygroscopic organic compound soluble in water and polar organic solvents. It is usually applied as a model compound for the study of the selective hydrogenation of polar alkynes[1]. The reaction was performed under MW irradiation in flow mode. The reactor cartridge (20 mL) was filled with Pd-loaded alumina balls (6 g) and with inert materials (3-8 mm Ø Pyrex glass balls 10g) resulting in a residual reactor volume of 10 mL. The liquid/gas reaction mixture ByD solution in ethanol (0.05 w/v%) and H_2 (7.5 mL/min) was pumped through the reactor (10 mL/min) under MW irradiation (350 W, reaction temperature 65 °C). The FlowSYNTH work station enables a precise monitoring of all key parameters. The reaction work up entails only solvent evaporation and direct GC-MS analysis (1 mg/mL EtOH/$CHCl_3$ 9:1).

TGA, SEM combined with EDX, XRPD, and TEM measurements

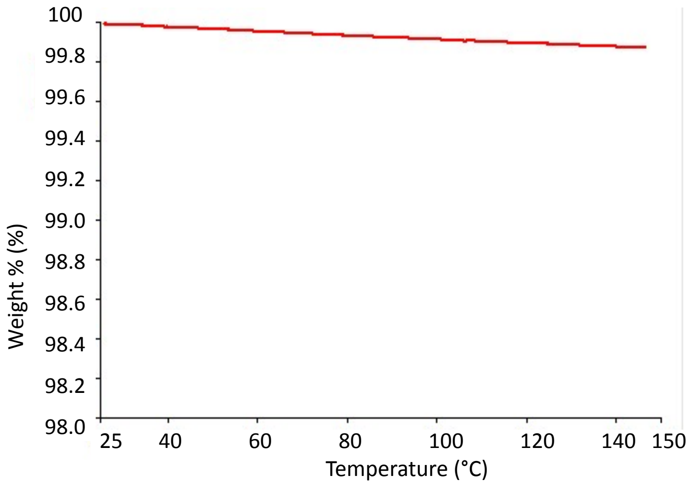

Figure SI-1. TGA analysis of the as-synthesised catalyst.

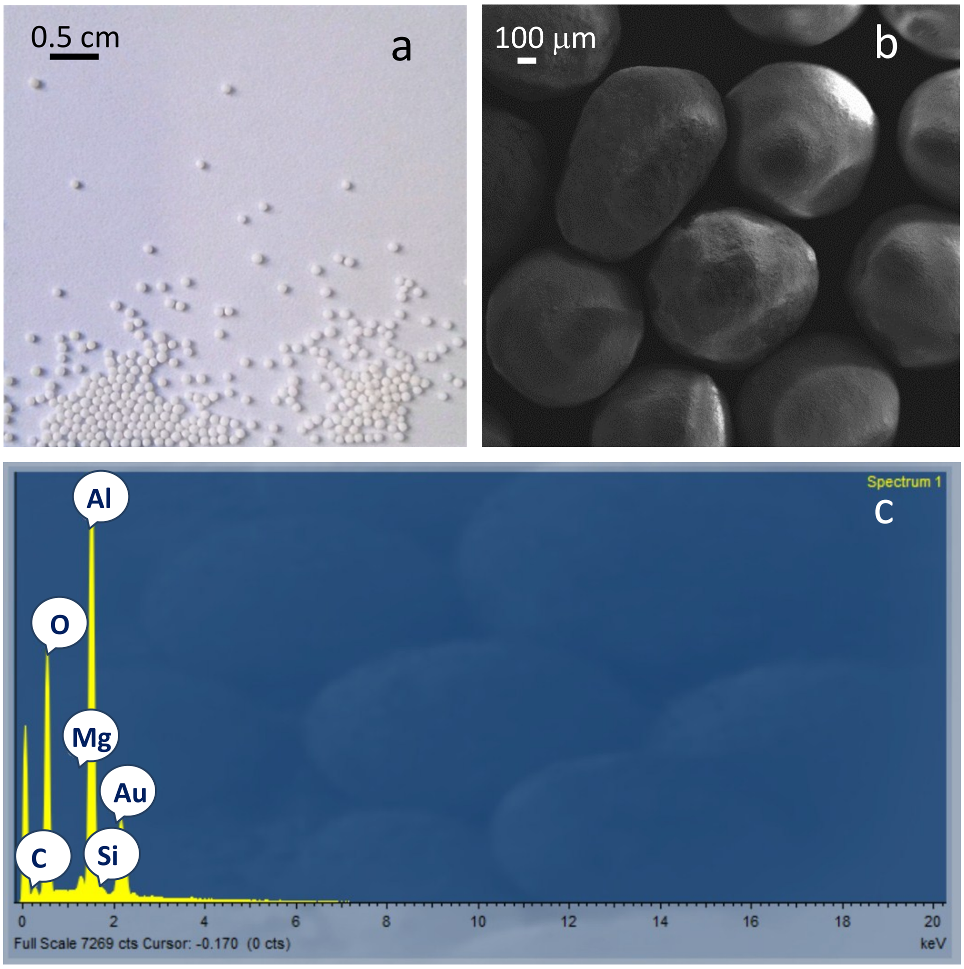

Figure SI-2. Image of the alumina-based balls employed as support (section a), SEM enlargement of the same balls and EDX spectrum (section c). Instrumental magnification 100×.

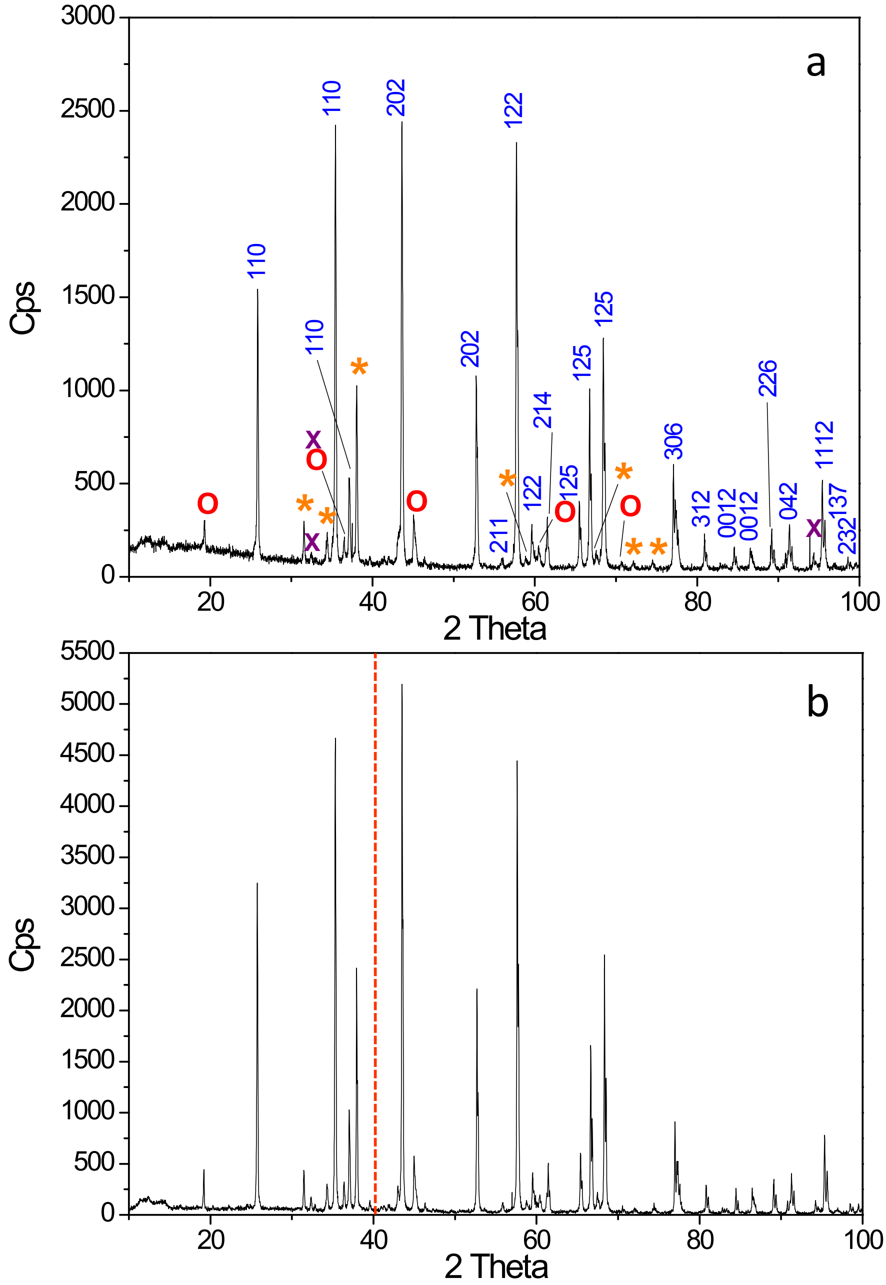

Figure SI-3. XRPD patterns of the alumina-based balls employed as support (section a) and of the Pd catalyst (section b). The peaks related to the rhombohedral Al_2O_3 corundum main phase (JCPDS file number 00-001-1296) are indexed in blue. The peaks related to the other observed crystalline phases have been marked with different colours. Orange symbols: orthorhombic $MgSiO_3$ (JCPDS file number 00-003-0519); Violet symbols: orthorhombic $MgSiO_4$ (JCPDS file number 00-034-0189) and red symbols: monoclinic Mg, Al oxide (JCPDS file number 00-010-0238).

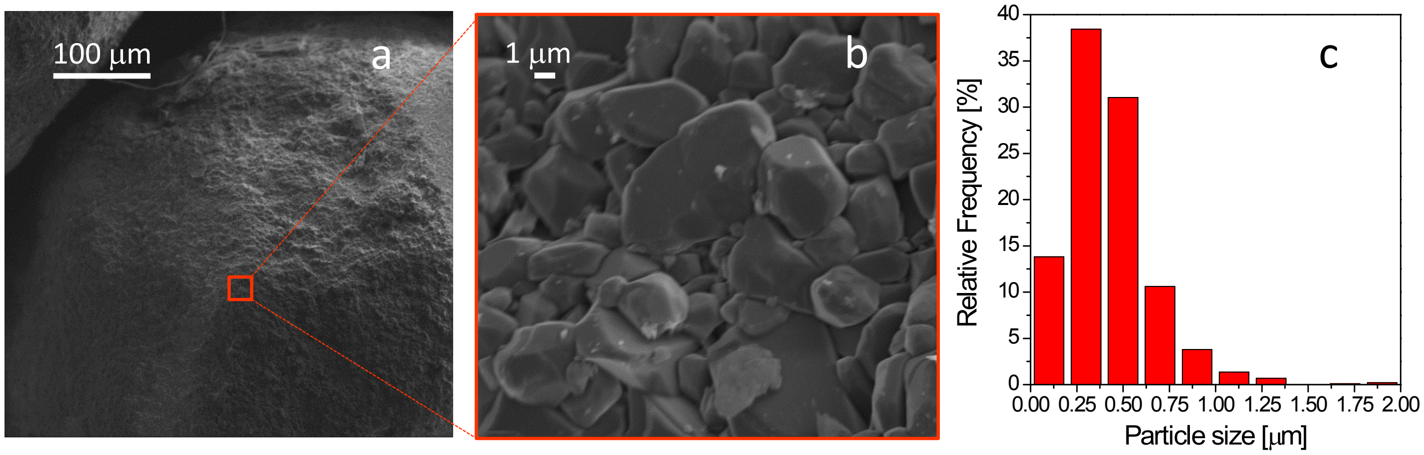

Figure SI-4. SEM image of the surface of the alumina-based balls employed as support (section a) zoom on the same surface at higher magnification (section b) and particle size distribution of the sintered particles constituting the balls. Instrumental magnification 500× and 10,000×, respectively.

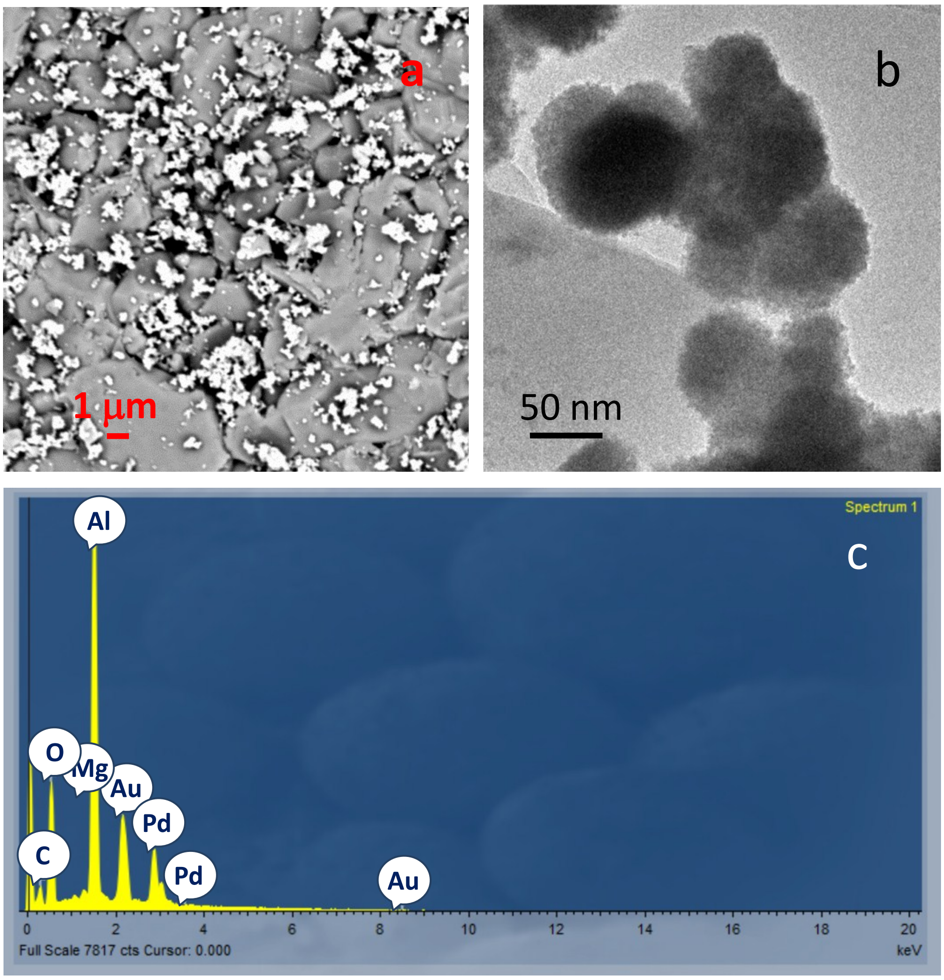

Figure SI-5. SEM image collected by using the BSE detector showing the surface of the catalyst (section a) and TEM image of the Pd catalyst (section b). EDX analysis performed on the sample shown in section a (section c). Instrumental magnification 10,000× and 50,000×, respectively.

In this case, the SEM images have been collected by means of the signal coming from the back scattered electrons in order to improve the contrast between the metal and the alumina surface. With this approach, the Pd particle agglomerates (whose composition is shown by the EDX analysis reported in section c) appeared white, whereas the surface of the ball was grey.

References

[1] a) M. Telkar, C. Rode, V. Rane, R. Jaganathan, R. Chaudhari, *Appl. Catal. A. Gen.* **2001**, *216,* 13–22; b) N. Semagina, E. Joannet, S. Parra, E. Sulman, A. Renken, L. Kiwi-Minsker, *Appl. Catal. A. Gen.* **2005**, *280*, 141–147.
